# Inter- and intra-chromosomal modulators of the *APOE* ɛ2 and ɛ4 effects on the Alzheimer’s disease risk

**DOI:** 10.1007/s11357-022-00617-0

**Published:** 2022-07-09

**Authors:** Alireza Nazarian, Ian Philipp, Irina Culminskaya, Liang He, Alexander M. Kulminski

**Affiliations:** Biodemography of Aging Research Unit, Social Science Research Institute, Duke University, Erwin Mill Building, 2024 W. Main St, Durham, NC 27705 USA

**Keywords:** Dementia, Aging, LD, Cox regression, Compound genotype, Genetic heterogeneity

## Abstract

The mechanisms of incomplete penetrance of risk-modifying impacts of apolipoprotein E (*APOE*) ε2 and ε4 alleles on Alzheimer’s disease (AD) have not been fully understood. We performed genome-wide analysis of differences in linkage disequilibrium (LD) patterns between 6,136 AD-affected and 10,555 AD-unaffected subjects from five independent studies to explore whether the association of the *APOE* ε2 allele (encoded by rs7412 polymorphism) and ε4 allele (encoded by rs429358 polymorphism) with AD was modulated by autosomal polymorphisms. The LD analysis identified 24 (mostly inter-chromosomal) and 57 (primarily intra-chromosomal) autosomal polymorphisms with significant differences in LD with either rs7412 or rs429358, respectively, between AD-affected and AD-unaffected subjects, indicating their potential modulatory roles. Our Cox regression analysis showed that minor alleles of four inter-chromosomal and ten intra-chromosomal polymorphisms exerted significant modulating effects on the ε2- and ε4-associated AD risks, respectively, and identified ε2-independent (rs2884183 polymorphism, 11q22.3) and ε4-independent (rs483082 polymorphism, 19q13.32) associations with AD. Our functional analysis highlighted ε2- and/or ε4-linked processes affecting the lipid and lipoprotein metabolism and cell junction organization which may contribute to AD pathogenesis. These findings provide insights into the ε2- and ε4-associated mechanisms of AD pathogenesis, underlying their incomplete penetrance.

**Supplementary information:**

The online version contains supplementary material available at 10.1007/s11357-022-00617-0.

## Introduction

The apolipoprotein E (*APOE*) gene is the strongest Alzheimer’s disease (AD)-associated genetic factor [1–3], which can explain 13.4% of phenotypic variance and 25.2% of genetic variance of AD [4]. Minor alleles of the exonic single-nucleotide polymorphisms (SNPs) rs429358 and rs7412 in the *APOE* gene encode the ε4 and ε2 alleles, respectively. The ε2 allele is considered as a protective factor against AD, whereas the ε4 allele is advocated to be a major variant predisposing to AD [3, 5].

The *APOE* gene encodes a lipoprotein mainly involved in lipid transfer and metabolism. Nevertheless, its functional impacts are not limited to lipid profile alterations and related vasculopathies [6]. The *APOE* involvement in AD pathogenesis has been widely studied, revealing various molecular and biological processes differentially impacted by different *APOE* alleles. For instance, the ε4 allele has been linked to increased production and decreased clearance of β-amyloid, stress-mediated increased tau hyperphosphorylation, accelerated cortical atrophy (e.g., in the medial temporal lobe), baseline neuronal hyperactivity (e.g., in the hippocampus), reduced cerebral glucose metabolism, damaged synaptic structure and function, increased cytoskeletal and mitochondrial dysfunction, and abnormal hippocampal neurogenesis [7].

Despite strong associations between *APOE* and AD, neither the ε2 nor ε4 allele is considered as a causal factor for AD development [5, 8–10]. Addressing the mechanisms of actions of the ε2 and ε4 alleles is essential for understanding AD pathogenesis and AD risk assessment. The complex regional interactions and haplotype structures in the *APOE* locus (19q13.3) have been emphasized by a growing body of studies [11–19]. These studies indicate the potential roles of nearby polymorphisms in modulating the impacts of the *APOE* alleles on AD risks in the form of haplotypes and combinations of genotypes (called compound genotypes). The analyses of haplotypes leverage the idea that AD can be affected by haplotypes driven by genetic linkage between nearby SNPs [20]. The functional linkage may drive, however, compound genotypes consisting of not only local but also distant variants [21].

In this study, we used a comprehensive approach to examine intra- (cis-acting) and inter- (trans-acting) chromosomal modulators of the impacts of the *APOE* rs7412 or rs429358 SNPs on the AD risk in the ε4- or ε2-negative sample. We leveraged samples of the AD-affected (*N* = 6,136) and unaffected (*N* = 10,555) subjects from five studies: (i) to perform a comparative analysis of LD between rs7412 or rs429358 and other autosomal SNPs in the human genome in the AD-affected and unaffected subjects, (ii) to examine AD risks for carriers of compound genotypes comprised of rs7412 or rs429358 and the identified intra- and inter-chromosomal SNPs in LD with them, and (iii) to identify biological functions and diseases enriched by genes harboring these SNPs.

## Methods

### Study participants

We used data on subjects of European ancestry from (Table S1): three National Institute on Aging (NIA) Alzheimer’s Disease Centers data (ADCs) from the Alzheimer’s Disease Genetics Consortium (ADGC) initiative [22], whole-genome sequencing (WGS) data from the Alzheimer’s Disease Sequencing Project (ADSP-WGS) [23, 24], Cardiovascular Health Study (CHS) [25], Framingham Heart Study (FHS) [26, 27], and NIA Late-Onset Alzheimer’s Disease Family Based Study (LOAD FBS) [28]. The ADSP-WGS’s subjects who were also present in other datasets were excluded to make datasets independent. The *APOE* genotypes were either directly reported by original studies (ADGC, ADSP-WGS, FHS) or were determined based on the rs429358 and rs7412 genotypes (CHS and LOAD FBS). The diagnoses of AD cases in the five analyzed datasets were mainly based on the neurologic exams [29, 30], and the AD status was reported either directly (ADGC, ADSP-WGS, FHS, LOAD FBS) or in the form of ICD-9 (International Classification of Disease codes, ninth revision) codes (CHS).

### Genotype data and quality control (QC)

We used whole-genome sequencing (ADSP-WGS) and genome-wide data from different array-based platforms (ADGC, CHS, FHS, LOAD FBS). SNPs were first imputed to harmonize them across analyzed datasets [31]. Low-quality data were excluded using *PLINK* [32] as follows: (1) SNPs and subjects with missing rates > 5%, (2) SNPs with minor allele frequencies (MAF) < 5%, (3) SNPs deviated from Hardy–Weinberg with *P* < 1E-06, and (4) SNPs, subjects, and/or families with Mendel error rates > 2% (in ADSP-WGS, FHS, and LOAD FBS which include families). In addition, imputed SNPs with *r*^2^ < 0.7 were filtered out (ADGC, CHS, FHS, LOAD FBS). Selecting SNPs presented at least in one study resulted in a set of 1,645,025 SNPs for the analysis.

### Two-stage LD analysis

#### Design

Our analyses were performed separately in stratified samples obtained by dividing each dataset into four groups based on the *APOE* genotypes and AD status. First, we determined ε4-negative (ε2ε2, ε2ε3, and ε3ε3 genotypes) and ε2-negative (ε4ε4, ε3ε4, and ε3ε3 genotypes) subsamples. Then, each subsample was divided into AD-affected and unaffected groups (herein referred to as AD and NAD groups, respectively). We evaluated LD between the *APOE* rs7412 or rs429358 SNP and each SNP in the genome in two stages.

#### Stage 1: LD analysis in individual and pooled datasets

We examined LD (i.e., *r* statistics) using the haplotype-based method [33–35] in each of the four selected subsamples in each dataset individually and combined. The statistically significant LD estimates were determined using a conservative chi-square test *χ*^*2*^ = *r*^*2*^*n* [35], where *n* is the number of subjects rather than gametes to address the uncertainty in inferring haplotypes from unphased genetic data [16, 18, 36, 37]. The variances of the *r* statistics were calculated using the asymptotic variance method detailed in [37]. The LD analysis was performed using *haplo.stats r* package [38].

Stage 1 provided two sets of SNPs in LD with the *APOE* SNPs in each subsample. The first set was generated following the discovery-replication strategy (herein referred to as replication set). In this case, SNPs were selected if their LD with the *APOE* SNP attained: (1) genome-wide (*P* < 5E-08) or suggestive-effect (5E-08 ≤ *P* < 5E-06) significance in any of the five datasets, which was considered as a discovery set, and (2) Bonferroni-adjusted *P* < 0.0125 (= 0.05/4, where 4 is the number of potential replication sets) in at least one of the other four datasets [31]. The second set included SNPs in significant LD with the *APOE* SNPs at genome-wide or suggestive significance in the pooled samples of all five datasets that were not in the replication set.

#### Stage 2: group-specific LD

We examined whether SNPs identified in stage 1 had group-specific LD by contrasting *r* between pooled AD and NAD groups, *Δr* = *r*_AD_-*r*_NAD_, using a permutation test [39, 40]. Significant *Δr* indicated SNPs in group-specific LD with rs7412 or rs429358. Bonferroni-adjusted thresholds, accounting for the number of tested SNPs, were used to identify significant findings.

### Analysis of the AD risk

For each group-specific SNP, survival-type analysis was performed to examine the impact of a compound genotype variable (CompG) on the AD risk. The CompG included four compound genotypes comprised of rs7412 or rs429358 genotypes and genotypes of a group-specific SNP (Table 1).

**Table 1:** Compound genotype constructed based on the genotypes at rs7412 or rs429358 and the identified group-specific SNPs

Genotype at rs7412 or rs429358	Genotype at the group-specific SNP	Compound genotype
0	0	CompG1
0	1 or 2	CompG2
1 or 2	0	CompG3
1 or 2	1 or 2	CompG4

Abbreviations: *SNP*, single-nucleotide polymorphism; *CompG*, compound genotype; 0, major allele homozygote; 1, heterozygote; 2, minor allele homozygote; *CompG1*, ε3ε3 subjects carrying major allele homozygotes of the SNP; *CompG2*, ε3ε3 subjects carrying at least one minor allele of the SNP; *CompG3*, ε2 carriers (i.e., ε2ε2 or ε2ε3 subjects in the rs7412 analysis) or ε4 carriers (i.e., ε4ε4 or ε3ε4 subjects in the rs429358 analysis) having major allele homozygotes of the SNP; *CompG4*, ε2 (rs7412 analysis) or ε4 (rs429358 analysis) carriers having at least one minor allele of the SNP

We fitted the Cox regression model (*coxme* and *survival* R packages [41, 42]), considering the age at onset of AD as a time variable. We used sex, the top five principal components of genetic data and ADC cohorts (in ADGC) as fixed-effects covariates, and family IDs (LOAD FBS, FHS, ADSP-WGS) as a random-effects covariate. The results from five datasets were combined through inverse-variance meta-analysis using *GWAMA* package [43]. The CompG1 compound genotype was the reference factor level. We used a chi-square test with one degree of freedom [44] to estimate the significance of the difference between the effect sizes for CompG3 and CompG4:

$${\chi }^{2}= \frac{{\left({b}_{CompG3}-{b}_{CompG4}\right)}^{2}}{{se}_{CompG3}^{2}+ {se}_{CompG4}^{2}}$$

where *b*_*CompG3*_ (*se*_*CompG3*_) and *b*_*CompG4*_ (*se*_*compG4*_) are the beta coefficients (standard errors) corresponding to the CompG3 and CompG4 genotypes in the Cox model, respectively. Significant findings were identified at the Bonferroni-adjusted levels correcting for the numbers of ε2- and ε4-associated group-specific SNPs.

### Functional enrichment analysis

The *Database for Annotation, Visualization and Integrated Discovery (DAVID)* [45] and *Metascape* [46] web tools were used to identify gene-enriched *REACTOME* pathways [47] and *DisGeNET* diseases [48]. The analysis was performed for genes harboring SNPs in group-specific LD with rs7412 or rs429358 separately. We used false discovery rate (FDR) adjusted significance cut off at *P*_FDR_ < 0.05 [49] to identify significantly enriched terms by two or more genes.

## Results

### SNPs in LD with rs7412 (*APOE* ε2 allele)

In stage 1, we found that 306 SNPs mapped to 27 loci were in LD with rs7412 at *P* < 5E-06 in the AD group (21 SNPs in 9 loci, Table S2), the NAD group (198 SNPs in 20 loci, Table S3), or both AD and NAD groups (87 SNPs, all in the *APOE* locus, Table S4). Of them, we identified LD of rs7412 with 58 SNPs not in the *APOE* locus (or other loci on chromosome 19) in the AD (19 SNPs in 8 loci) or NAD (39 SNPs in 19 loci) groups. For most SNPs, 219 of 306, the magnitudes of LD (i.e., |*r*|) were smaller in the pooled AD than NAD group (181 of 248 SNPs in the *APOE* locus and 38 of 58 inter-chromosomal SNPs). We also observed that the *r* signs were the same in these two groups for 272 of 306 SNPs.

**Fig. 1:**
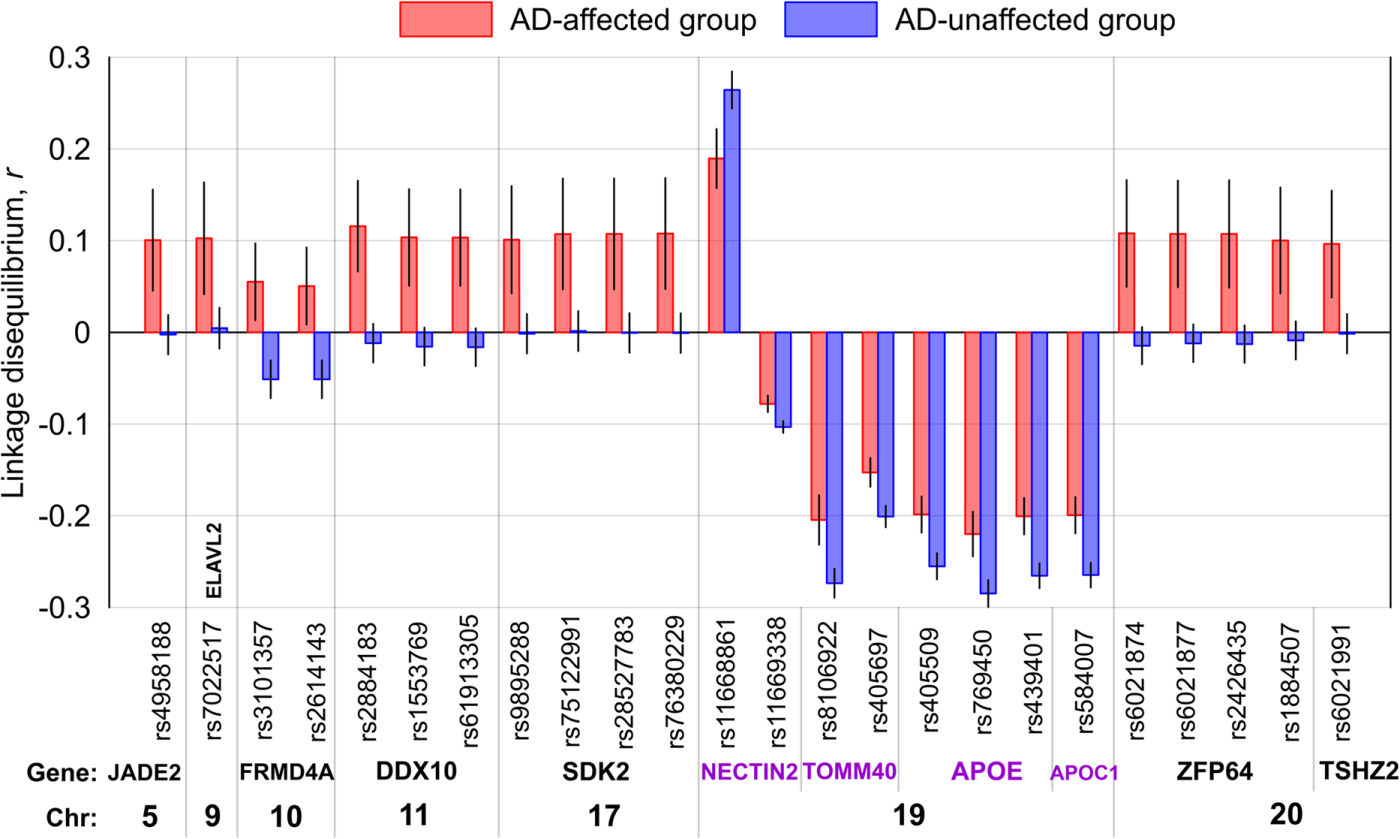
Linkage disequilibrium *r* between the identified group-specific SNPs and rs7412 in the ε4-negative sample of all five datasets combined. The *x*-axis shows SNP identifiers, genes harboring these SNPs, and chromosomes. Red boxes: Alzheimer’s disease-affected group (AD). Blue boxes: Alzheimer’s disease-unaffected group (NAD). The vertical lines show 95% confidence intervals

In stage 2, we found 24 SNPs (Table S5) having group-specific LD with rs7412 at a Bonferroni-adjusted significance *P* < 1.63E-04 (= 0.05/306). Of them, 16 SNPs were mapped to 6 non-*APOE* loci. All of them were identified in the pooled sample of either the AD (14 SNPs) or NAD (2 SNPs) group. LD estimates for 14 of these 16 SNPs were characterized by opposite signs of *r* in these groups (Fig. 1). Also, 15 of them had larger magnitudes of *r* in the AD group than NAD group. The remaining 8 SNPs were in the *APOE* locus, of which rs11669338 (*NECTIN2*) attained significance only in NAD group, whereas all the others were significant in both groups. All 8 SNPs had the same signs of *r* in the AD and NAD groups, whose magnitudes were smaller in the AD than NAD group (Fig. 1).

### SNPs in LD with rs429358 (*APOE* ε4 allele)

In stage 1, we found that rs429358 was in LD with 801 SNPs (143 loci) at *P* < 5E-06 in the AD group (301 SNP in 73 loci, Table S6), the NAD group (351 SNP in 81 loci, Table S7), or both AD and NAD groups (149 SNP; all in the *APOE* locus, except 2 SNPs, Table S8). In the AD and NAD groups, we identified LD of rs429358 with 159 (72 loci) and 344 (80 loci) SNPs not in the *APOE* region, respectively, totaling 503 SNPs. Of all 505 SNPs (154 loci) not in the *APOE* locus in AD, NAD, and AD&NAD groups, one locus harboring *FXYD5* and *FAM187B* genes (11 SNPs, NAD group) was on chromosome 19, and the other 494 SNPs (153 loci) were not on chromosome 19. The LD magnitudes were smaller in the pooled AD than NAD group for 370 of 801 SNPs (26 of 296 SNPs in the *APOE* locus and 344 of 505 SNPs in the non-*APOE* loci). The *r* signs were the same in these two groups for 711 of 801 SNPs.

**Fig. 2:**
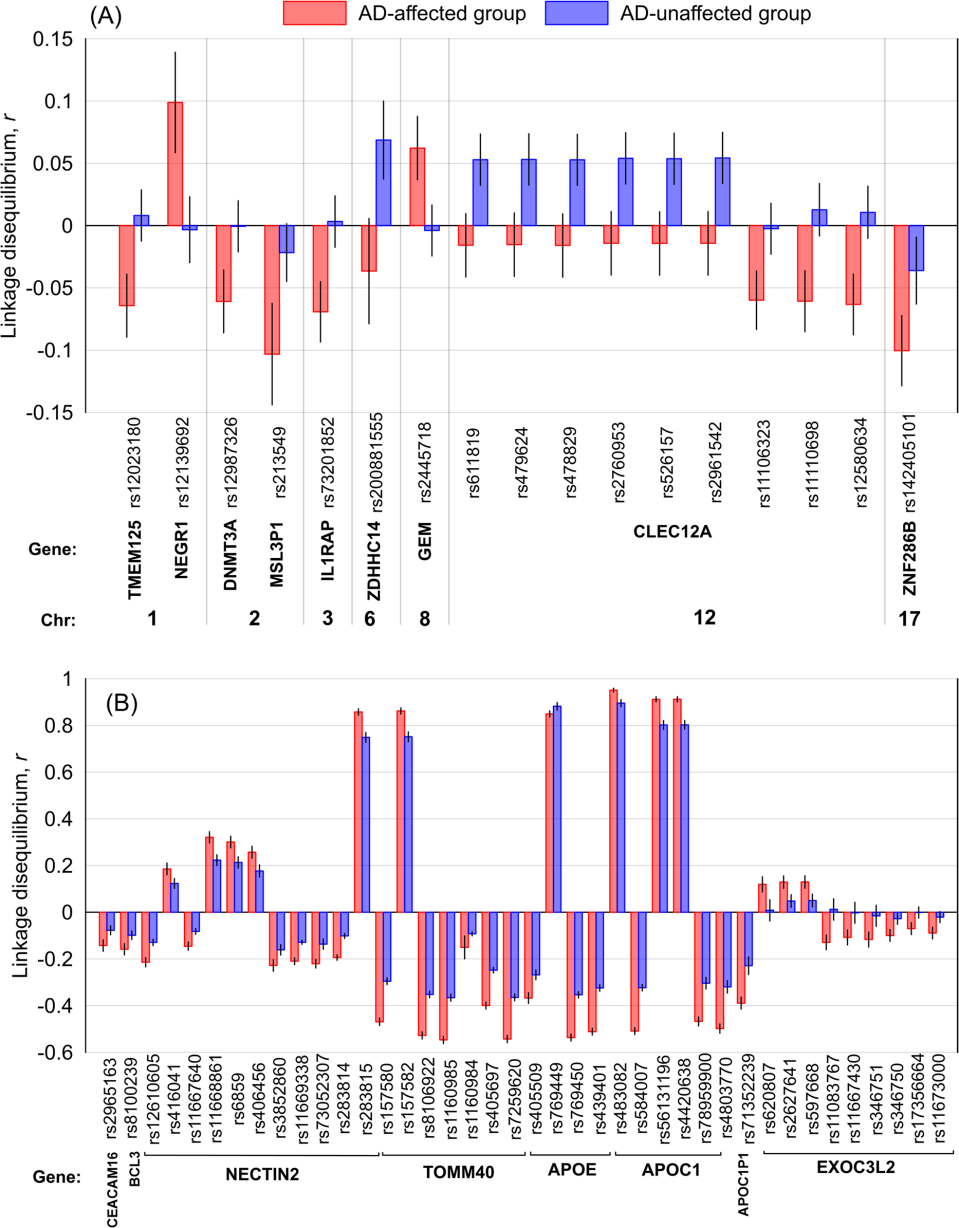
Linkage disequilibrium (LD) *r* between the identified group-specific SNPs and rs429358 in the ε2-negative sample of all five datasets combined. (**A**) LD for inter-chromosomal SNPs, i.e., SNPs not on chromosome 19. (**B**) LD for intra-chromosomal SNPs. The *x*-axis shows SNP identifiers, genes harboring these SNPs, and chromosomes in **A**. Red boxes: Alzheimer’s disease-affected group (AD). Blue boxes: Alzheimer’s disease-unaffected group (NAD). The vertical lines show 95% confidence intervals

In stage 2, we identified 57 SNPs with group-specific LD at a Bonferroni-adjusted significance *P* < 6.24E-05 (= 0.05/801). As seen in Table S9, 17 of 57 SNPs are mapped to 11 non-*APOE* loci. All of them were identified in the pooled sample of either the AD (10 SNPs) or NAD (7 SNPs) group. The magnitudes of *r* were larger in the pooled AD than NAD sample for SNPs whose significant LD was identified in the AD group and vice versa. The *r* signs for 13 of these 17 SNPs were opposite in these AD and NAD samples. The other 40 SNPs were located in the *APOE* locus. Magnitudes of *r* for all SNPs, except rs769449 (*APOE*), were larger in the pooled AD than NAD sample. For all SNPs, except rs11083767 (*EXOC3L2*), the *r* signs were the same in these AD and NAD samples (Fig. 2).

### AD risk for carriers of compound genotypes

**Fig. 3:**
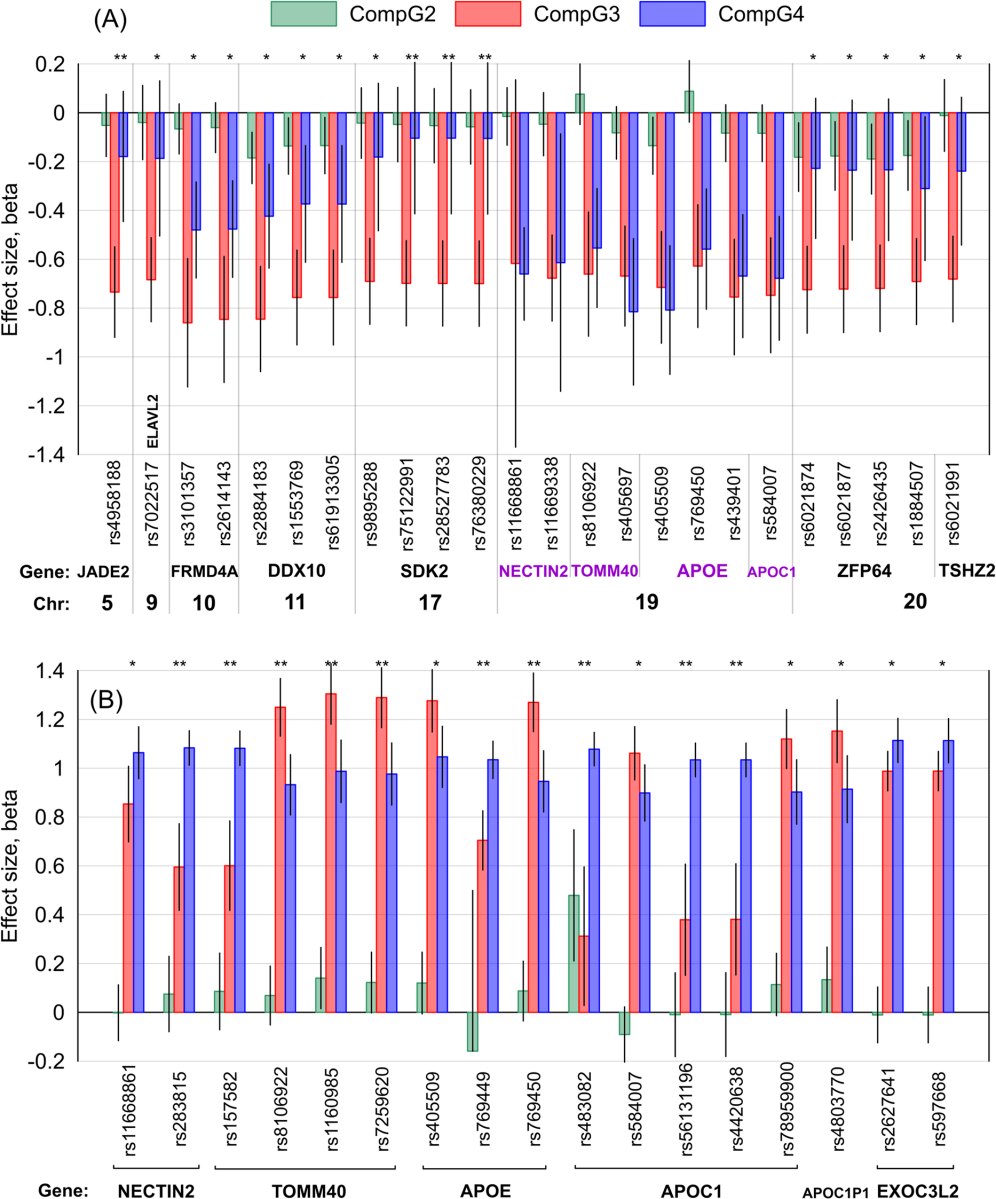
The results of the meta-analysis of the associations of compound genotypes comprised of SNPs (shown on the *x*-axis) in group-specific linkage disequilibrium with (**A**) rs7412 in the ε4-negative sample or (**B**) rs429358 in the ε2-negative sample with the Alzheimer’s disease risk. CompG2 (green) indicates ε3ε3 subjects carrying at least one minor allele of the SNP; CompG3 (red) denotes (**A**) ε2 or (**B**) ε4 carriers having major allele homozygotes of the SNP; CompG4 (blue) indicates (**A**) ε2 or (**B**) ε4 carriers having at least one minor allele of the SNP. CompG1 indicating the ε3ε3 subjects carrying major allele homozygotes of the SNP was the reference. Black vertical lines show 95% confidence intervals (negative direction for rs769449 was truncated for better resolution). The *x*-axis shows SNP identifiers, genes harboring these SNPs, and chromosomes. One asterisk (*) indicates nominally significant differences in the effects between CompG3 and CompG4 at (**A**) 2.08E-03 ≤ *P* < 0.05 and (**B**) 8.77E-04 ≤ *P* < 0.05. Two asterisks (**) indicate Bonferroni-adjusted significance in those differences at (**A**) *P* < 2.08E-03 and (**B**) *P* < 8.77E-04. No asterisk indicates non-significant differences in (**A**). **B** shows only 17 group-specific SNPs for which the differences in the effects between CompG3 and CompG4 attained *P* < 0.05

We performed Cox regression analysis to examine the impact of compound genotypes comprised of a group-specific SNP and either rs7412 (Tables 2 and S10, Fig. 3A) or rs429358 (Tables 2 and S11, Fig. 3B) on the AD risk. An advantage of using compound genotypes is that we can explicitly examine the effect of a minor allele of a group-specific SNP independently of the effect of the ε2 or ε4 allele (CompG2), the impact of the ε2 or ε4 allele independently of the minor allele of that SNP (CompG3), and the combined effects of these minor alleles (CompG4) in the same model with the same reference genotype (CompG1) (Table 1).

**Table 2:** Bonferroni-adjusted significant results from the survival-type meta-analysis of compound genotype (CompG) associations with Alzheimer’s disease risk using SNPs in group-specific LD with rs7412 or rs429358

Group-specific SNPs							CompG2				CompG3				CompG4				CompG3 vs. CompG4		
CHR	GENE	SNP	POS	EA	EAF	N	BETA	SE	*P* value	Effects	BETA	SE	*P* value	Effects	BETA	SE	*P* value	Effects	|Beta|.diff	χ^2^	*P* value
rs7412 analysis																					
5q31.1	*JADE2*	rs4958188	134,504,121	T	0.106	6853	-0.052	0.065	4.27E-01	- - + + -	-0.734	0.095	**9.05E-15**	- - + - -	-0.179	0.136	1.88E-01	+ - - + -	-0.555	11.242	**8.00E-04**
11q22.3	*DDX10*	rs2884183	109,127,412	T	0.212	6849	-0.185	0.054	**5.46E-04**	- - - - -	-0.845	0.110	**1.39E-14**	- - - - -	-0.423	0.109	**9.72E-05**	- - - - -	-0.422	7.464	6.29E-03
17q25.1	*SDK2*	rs75122991	73,639,797	G	0.068	6853	-0.048	0.078	5.35E-01	+ - + + -	-0.698	0.089	**4.95E-15**	- - - - -	-0.104	0.158	5.12E-01	+ - + ? -	-0.595	10.732	**1.05E-03**
17q25.1	*SDK2*	rs28527783	73,640,957	T	0.069	6854	-0.053	0.078	4.95E-01	+ - + + -	-0.699	0.089	**4.66E-15**	- - - - -	-0.104	0.158	5.10E-01	+ - + ? -	-0.595	10.741	**1.05E-03**
17q25.1	*SDK2*	rs76380229	73,643,396	T	0.068	6852	-0.058	0.078	4.58E-01	+ - + + -	-0.700	0.089	**4.42E-15**	- - - - -	-0.105	0.158	5.07E-01	+ - + ? -	-0.595	10.731	**1.05E-03**
rs429358 analysis																					
19q13.32	*NECTIN2*	rs283815	44,887,076	G	0.297	9935	0.075	0.079	3.42E-01	+ + ? + -	0.595	0.091	**5.32E-11**	+ + ? + +	1.083	0.036	**1.09E-197**	+ + ? + +	0.488	25.030	**5.64E-07**
19q13.32	*TOMM40*	rs157582	44,892,962	T	0.297	9933	0.086	0.080	2.85E-01	+ - ? + -	0.601	0.093	**1.33E-10**	+ + ? + +	1.082	0.036	**5.12E-198**	+ + ? + +	0.481	23.079	**1.56E-06**
19q13.32	*TOMM40*	rs8106922	44,898,409	G	0.372	9919	0.069	0.061	2.62E-01	- + ? - -	1.249	0.060	**2.04E-95**	+ + ? + +	0.932	0.063	**1.88E-49**	+ + ? + +	-0.317	13.241	**2.74E-04**
19q13.32	*TOMM40*	rs1160985	44,900,155	T	0.394	9935	0.140	0.064	2.85E-02	+ + ? + -	1.304	0.063	**1.43E-94**	+ + ? + +	0.987	0.065	**1.01E-51**	+ + ? + +	-0.317	12.202	**4.78E-04**
19q13.32	*TOMM40*	rs7259620	44,904,531	A	0.391	9935	0.122	0.064	5.50E-02	+ + ? - -	1.289	0.063	**1.27E-93**	+ + ? + +	0.976	0.065	**3.60E-51**	+ + ? + +	-0.313	11.993	**5.34E-04**
19q13.32	*APOE*	rs769449	44,906,745	A	0.198	7860	-0.159	0.336	6.36E-01	? - ? ? ?	0.704	0.062	**7.57E-30**	? + ? + +	1.035	0.039	**1.40E-154**	? + ? + +	0.330	20.312	**6.58E-06**
19q13.32	*APOE*	rs769450	44,907,187	A	0.376	9933	0.087	0.062	1.62E-01	- + ? - -	1.270	0.061	**2.09E-95**	+ + ? + +	0.946	0.064	**1.25E-49**	+ + ? + +	-0.324	13.387	**2.53E-04**
19q13.32	*APOC1*	rs483082	44,912,921	T	0.276	9933	0.479	0.137	**4.75E-04**	+ - ? + -	0.312	0.145	3.12E-02	+ + ? ? +	1.078	0.035	**6.12E-209**	+ + ? + +	0.766	26.454	**2.70E-07**
19q13.32	*APOC1*	rs56131196	44,919,589	A	0.328	7667	-0.009	0.088	9.14E-01	+ - + ? -	0.379	0.116	1.10E-03	+ + - ? +	1.034	0.035	**1.02E-189**	+ + + ? +	0.655	29.080	**6.94E-08**
19q13.32	*APOC1*	rs4420638	44,919,689	G	0.328	7660	-0.009	0.088	9.18E-01	+ - + ? -	0.381	0.116	1.05E-03	+ + - ? +	1.034	0.035	**1.63E-189**	+ + + ? +	0.653	28.924	**7.53E-08**

Abbreviations: *SNP*, single-nucleotide polymorphism; *CHR*, chromosomal region (i.e., cytogenetic band); *POS*, SNP position based on Human Genome version 38 (hg38); *EA*, effect allele; *EAF*, effect allele frequency; *N*, number of subjects; *BETA* and *SE*, effect size and its standard error; *effects*, directions of effects in LOAD FBS, ADGC, FHS, CHS, and ADSP-WGS datasets, respectively; *|Beta|.diff*, the difference in absolute values of effect sizes of CompG4 and CompG3 levels; χ.^2^, chi-square statistic corresponding to the comparison of effect sizes of CompG4 and CompG3 levels

#### ***AD risk for carriers of 24 rs7412-bearing compound genotypes (Tables ******2****** and S10, Fig. ******3******A)***

Our analysis showed that none of eight CompG2 genotypes bearing SNPs from the *APOE* locus attained Bonferroni-adjusted significance *P*_Bε2_ = 2.08E-03 (= 0.05/24), although rs405509 minor allele was beneficially associated with AD, independently of ε2, at nominal significance *P* = 0.0238. In contrast, six of 16 CompG2 genotypes comprised of rs7412 and non-*APOE* locus SNPs were beneficially associated with AD at the nominal significance (*P*_Bε2_ ≤ *P* < 0.05). For one CompG2, we observed beneficial association of rs2884183 minor allele (11q22.3, *DDX10*) with AD at *P* < *P*_Bε2_ independently of the ε2 allele.

All CompG3 genotypes were beneficially associated with AD (although non-significantly for rs11668861) because of the leading role of the ε2 allele and the lack of minor alleles of the group-specific SNPs. Also, regardless of the significance, all CompG4 genotypes were beneficially associated with AD risk, with 10 of them (seven in the *APOE* locus) reaching *P* < *P*_Bε2_. For all 16 group-specific inter-chromosomal SNPs, the effects for CompG4 were smaller in magnitude than those for CompG3 either at the nominal (12 SNPs) or *P* < *P*_Bε2_ (four SNPs) significance (Fig. 3A).

#### ***AD risk for carriers of 57 rs429358-bearing compound genotypes (Tables ******2****** and S11, Fig. ******3******B)***

We found that one of 40 intra-chromosomal CompG2 genotypes comprised of rs483082 (*APOC1*) and rs429358 was adversely associated with AD risk independently of the ε4 allele at Bonferroni-adjusted significance *P*_Bε4_ = 8.77E-04 (= 0.05/57). None of 17 CompG2 with inter-chromosomal SNPs attained *P* < *P*_Bε4_.

Each of 57 CompG3 and CompG4 genotypes was adversely associated with the AD risk. None of the differences in the effects between them attained *P* < *P*_Bε4_ for inter-chromosomal SNPs. In contrast, we identified seven (*P*_Bε4_ ≤ *P* < 0.05) and 10 (*P* < *P*_Bε4_) differences in the effects between CompG3 and CompG4 for SNPs within the *APOE* locus (Fig. 3B).

### Biological functions and diseases

Our analysis was performed for 11 and 19 genes harboring SNPs in group-specific LD with ε2-encoding rs7412 and ε4-encoding rs429358, respectively. We found that 7 and 4 *REACTOME* pathways were enriched at *P* < 0.05 using genes from the ε2 (Fig. S1) and ε4 (Fig. S2) sets, respectively. Four of them, i.e., “plasma lipoprotein assembly,” “plasma lipoprotein clearance,” “NR1H3 and NR1H2 regulate gene expression linked to cholesterol transport and efflux,” and “NR1H2 and NR1H3-mediated signaling,” were enriched in both ε2 and ε4 sets. Three pathways, however, were ε2-specific, including “cell–cell junction organization,” “plasma lipoprotein assembly, remodeling, and clearance,” and “cell junction organization.” There were no enriched ε4-specific pathways.

Disease annotations (Tables S12 and S13) included 14 terms that were enriched at *P*_FDR_ < 0.05 by both the ε2 and ε4 gene sets. They were mainly related to neurological diseases (e.g., AD and other dementia phenotypes, memory performance, mild cognitive disorder, and primary progressive aphasia), serum lipid traits (e.g., dyslipoproteinemias, serum low-density lipoprotein (LDL) cholesterol measurement, and serum total cholesterol measurement), serum albumin measurement, and C-reactive protein measurement.

Seven terms were only enriched in the ε4 set at *P*_FDR_ < 0.05 (Table S13) which included mental deterioration, atherogenesis, triglycerides measurement, and high-density lipoprotein measurement as well as multiple hematological and immune system-related terms (i.e., autoantibody measurement, acute monocytic leukemia, and peripheral T-cell lymphoma).

## Discussion

Our comprehensive approach examining intra- and inter-chromosomal modulators of the impacts of the *APOE* rs7412 or rs429358 SNP encoding the ε2 or ε4 allele on the AD risk provided four insights.

First, we identified 306 (27 loci) and 801 (143 loci) SNPs in LD with rs7412 and rs429358, respectively, at genome-wide (*P* < 5E-08) or suggestive-effect (5E-08 ≤ *P* < 5E-06) significance in AD, NAD, or both groups. Of them, 58 (27 loci) and 505 (154 loci) SNPs were not on *APOE* locus, indicating potential inter-chromosomal modulators of the impacts of the ε2 or ε4 allele on the AD risk.

Second, among these SNPs, we found significant differences in LD between AD and NAD groups for 24 (16 inter-chromosomal SNPs in 6 loci) and 57 (17 inter-chromosomal SNPs in 11 loci) SNPs with rs7412 and rs429358, respectively, at the Bonferroni-adjusted significance level (Figs. 1 and 2, and Tables S5 and S9). This finding strongly supports modulating roles of the intra- and inter-chromosomal SNPs on the impacts of the ε2 or ε4 allele on the AD risk, predominantly tailored to either AD-affected or unaffected subjects.

Third, Cox regression analysis identified Bonferroni-adjusted associations of minor alleles of rs2884183 (11q22.3, *DDX10*) and rs483082 (19q13.32, *APOC1*) with decreased and increased AD risk independently of the ε2 and ε4 alleles, respectively (Table 2).

Fourth, Cox regression analysis revealed that the beneficial and adverse effects of the ε2 and ε4 alleles, respectively, on the AD risks were significantly modulated by other SNPs, and that this modulation was fundamentally different for these alleles. Specifically, the beneficial effect of the ε2 allele was decreased by minor alleles of all 16 group-specific inter-chromosomal SNPs (with a significant decrease at Bonferroni-adjusted level for variants mapped to *JADE2* and *SDK2* genes) (Fig. 3A). In contrast, the adverse effect of the ε4 allele was significantly modulated by ten *APOE* locus (intra-chromosomal) SNPs; the ε4 impact was weakened by minor alleles of four SNPs mapped to *TOMM40* and *APOE* genes and major alleles of six SNPs mapped to *NECTIN2*, *TOMM40*, *APOE*, and *APOC1* genes (Fig. 3B).

The *APOE* locus-specific LD patterns corroborated our previous findings observed for SNP pairs [18] and triples [17]. However, according to the GWAS catalog [50], none of the identified 33 inter-chromosomal group-specific SNPs has been associated with AD or AD-related pathologies (e.g., amyloid plaque) in previous GWAS at genome-wide or suggestive significance. Rs1884507 (*ZFP64*, in LD with rs7412) and rs12139692 (*NEGR1*, in LD with rs429358) were associated with triglycerides [51] and intelligence [52], respectively, at *P* < 5E-08. Other SNPs mapped to *FRMD4A* [53] and *NEGR1* [54] have been previously associated with AD at genome-wide and suggestive significance, respectively. In addition, several SNPs mapped to the *JADE2*, *FRMD4A*, *DDX10*, *SDK2*, *ZFP64*, *TSHZ2*, *ZDHHC14*, *NEGR1*, and *SLC5A8* genes, in interaction with SNPs in the other non-*APOE*-locus genes, were associated with AD-related brain pathologies such as diffuse amyloid plaque, PHF-tau, and neurofibrillary tangles at *P* < 5E-08 [55]. Also, an *IL1RAP* variant was previously associated with amyloid plaque accumulation rate at *P* < 5E-08 [56]. Additionally, SNPs mapped to *JADE2*, *ELAVL2*, and *TSHZ2* have been associated with educational attainment [57] and those mapped to *ELAVL2* and *NEGR1* with intelligence and general cognitive ability [58, 59].

Next, we discuss *JADE2* and *SDK2* genes harboring inter-chromosomal SNPs, which significantly modulate the effects of the ε2 allele on AD risk (Table 2). *JADE2* is involved in ubiquitination of histone demethylase *LSD1* [60] and may play roles in the *LSD1*-mediated regulation of neurogenesis and myogenesis [61, 62]. *LSD1* is required for neuronal survival and was implicated in tau-induced neurodegeneration in AD and frontotemporal dementia [63, 64]. Additionally, *JADE2* (alias *PHF15*) may regulate the microglial inflammatory response [65].

*SDK2* is involved in lamina-specific synaptic connections which are essential to form neuronal circuits in retina that detect motion [66]. Visual impairments including motion detection abnormalities have been reported in AD [67] and Huntington’s disease [68]. Also, visual working memory (i.e., object identification and location recall) was previously associated with the ε4 allele and β-amyloid accumulation [69].

We also highlight *DDX10* gene harboring rs2884183, which is associated with AD risk independently of ε2 (Table 2). The RNA helicase *DDX10* affects ribosome assembly and modulates α-synuclein toxicity [70]. α-Synuclein may synergistically interact with β-amyloid and tau protein to promote their accumulation [71] and may be involved in the pathogenesis of AD in addition to synucleinopathie (e.g., Parkinson’s disease) [72, 73]. *DDX10* may also affect ovarian senescence [74].

Our enrichment analysis of biological functions (Figs. S1 and S2) suggested that group-specific LD with rs7412 or rs429358 entails SNPs in genes, which are involved in lipid and lipoprotein metabolism. Additionally, LD with rs7412 entails SNPs in genes, which may contribute to cell junction organization. These biological processes have been implicated in AD pathogenesis [31, 75–79]. The disease enrichment analysis (Tables S12 and S13) mostly highlighted the enrichment of AD, dementia phenotypes, and other neurological diseases as well serum lipid traits in both the ε2 and ε4 gene sets. In addition, multiple lipid traits and neurological and immune system-related disorders were enriched in the ε4 gene set.

Investigating the impacts of group-specific SNPs on gene expression revealed that several SNPs in LD with rs7412 (Table S5), including rs11668861 (*NECTIN2*), rs6021874, rs6021877, rs2426435, and rs1884507 (*ZFP64*), are in LD (*P* < 0.0001 in the CEU population of Utah Residents with Northern and Western European Ancestry [80]) with expression quantitative trait loci (eQTLs) whose minor alleles increase *NECTIN2* and *ZFP64* expressions in the brain tissue (Table S14). Also, among SNPs in group-specific LD with rs429358 (Table S9), SNPs mapped to *CLEC12A* (rs611819, rs479624, rs478829, rs2760953, rs526157, and rs2961542) and *NECTIN2* (rs416041, rs11668861, rs6859, rs406456, and rs3852860) are in LD (*P* < 0.0001 [80]) with eQTLs altering the expressions of these two genes. In addition, rs4803770 and rs71352239 (*APOC1P1*) are themselves eQTLs for this gene whose minor alleles decrease *APOC1P1* expression in the brain tissue (Table S14) [81]. In addition, the transcription factor-binding sites (TFBS) enrichment [46] shows that that *JADE2*, *ELAVL2*, *FRMD4A*, and *APOC1* genes (harboring ε2 group-specific SNPs) have a common TFBS motif corresponding to *RXRB* within ± 2 kb of their transcription starting sites (*P* < 2.00E-06 and *P*_FDR_ < 5.01E-03) [82]. Also, *TMEM125*, *DNMT3A*, *ZDHHC14*, and *BCL3* genes (harboring ε4 group-specific SNPs) share a TFBS motif corresponding to *SP3* within ± 2 kb of their transcription starting sites (*P* < 1.58E-05 and *P*_FDR_ < 2.51E-02) [82].

Despite the rigor, this study has limitations. The first is that GWAS datasets do not provide phased genetic data, and therefore, probabilistic estimates of haplotypes may adversely impact the power of LD analyses. Second, due to the small frequency of the ε2 allele in the general population, the LD analysis of rs7412 may not have optimal statistical power, particularly in the AD-affected group because of the protective role of the ε2 allele against AD. Third, because genotypes were available from WGS in ADSP and genome-wide arrays in the other datasets, we imputed SNPs to harmonize them across all five datasets. Imputation generally results in less accurate genotype calls compared with WGS, particularly in genomic regions with low coverage on the arrays. Low imputation quality may adversely impact the results of the analyses. Although we excluded SNPs with imputation quality of *r*^2^ < 0.7 to offset the impacts of potential inaccuracies, replication of the results using directly genotyped SNPs could add robustness to our findings. Fourth, while the Cox regression analysis of genetic associations using AAO of a complex trait provides higher statistical power than the logistic regression analysis of the case–control status [83], we acknowledge limited abilities in determining exact AAO due to slow progression of AD. For instance, AD is not usually diagnosed when the brain pathologies start to develop years before clinical manifestations. Fifth, the small number of genes may affect the accuracy of the functional enrichment analysis. Finally, further stratifying of the AD group based on the pathological information on AD sub-phenotypes would provide valuable insights into the genetic heterogeneity of AD. Also, including subjects with mild cognitive impairment (MCI) in LD analyses as a separate stratum may help to identify *APOE* allele-dependent genetic factors contributing to MCI progression to AD. Such additional stratifications would require large datasets with more comprehensive clinical and pathological data.

## Conclusion

Our comprehensive analysis provides compelling evidence that intra- and inter-chromosomal variants can modulate the impacts of the ε2 and ε4 alleles on the AD risk. The survival-type analysis robustly shows predominant modulating roles of the inter-chromosomal SNPs for the ε2 allele and the *APOE-*region SNPs for the ε4 allele. We identified two variants in *DDX10* (11q22.3) and *APOC1* (19q13.32) genes with beneficial and adverse associations with AD risk independently of the ε2 and ε4 alleles, respectively. Functional enrichment analysis highlighted ε2- and/or ε4-linked processes involved in lipid and lipoprotein metabolism and cell junction organization which have been implicated in AD pathogenesis. Our results advance the understanding of the mechanisms of AD pathogenesis and help improve the accuracy of AD risk assessment.

## Supplementary information

Below is the link to the electronic supplementary material.

Supplementary file1 (PDF 319 KB)

Supplementary file2 (XLSX 30 KB)

Supplementary file3 (XLSX 155 KB)

Supplementary file4 (XLSX 75 KB)

Supplementary file5 (XLSX 33 KB)

Supplementary file6 (XLSX 230 KB)

Supplementary file7 (XLSX 269 KB)

Supplementary file8 (XLSX 117 KB)

Supplementary file9 (XLSX 54 KB)

Supplementary file10 (XLSX 21 KB)

Supplementary file11 (XLSX 32 KB)

Supplementary file12 (XLSX 11 KB)

Supplementary file13 (XLSX 12 KB)

Supplementary file14 (XLSX 11.8 KB)

## Data Availability

Data used in this study can be obtained from dbGaP (https://www.ncbi.nlm.nih.gov/gap/) and NIAGADS (https://www.niagads.org/adsp/content/home).
